# A prospective study on elective surgical inpatient satisfaction with perioperative anaesthesia service at Ayder comprehensive specialized hospital, Mekelle, Ethiopia

**DOI:** 10.1186/s12871-019-0696-8

**Published:** 2019-04-01

**Authors:** Kore Menjie Benwu, Hagos Gebregziabher Gebremedhin

**Affiliations:** Mekelle University College of Health Science Department of Anesthesia, Po-box 1871, Mekelle, Ethiopia

**Keywords:** Patient satisfaction, Perioperative anaesthesia service, Associated factors, Operation

## Abstract

**Background:**

Patient satisfaction is a subjective and challenging perception, linking physical, expressive, psychological, societal, and cultural factors. Dissatisfaction arises if the patient feels an inconsistency between expected and delivered care. Usually health care satisfactions are very high and according to many studies levels of satisfaction are above 85% and patient’s satisfaction in terms of anesthesia is not very different. The aim of this study was to assess patient’s satisfaction with perioperative anesthesia service and associated factors.

**Methods:**

Institution-based cross-sectional study was conducted from December to January, 2017/8 at the Ayder Comprehensive Specialize Hospital. The data were collected using structured interviewer-administered questionnaire prepared to collect data on demographic character of the patients, determinant factors which could affect the patient satisfaction level on anesthesia service. Epi Info version 6 was used to record the data and SPSS version 20 was used for the analysis. Descriptive statistics were used to explore the socio-demographic characteristics of patients; factors possibly related to satisfaction level and overall satisfaction were summarized as frequencies and percentages.

**Results:**

One hundred twenty consecutive patients were originally enrolled in the study that took over 1 Month. The overall proportion of patients who satisfied with anaesthesia services was 88.33%. Nausea and vomiting, pain, shortness of breath and cold were factors which affected patient satisfaction negatively.

**Conclusion and recommendation:**

Compared with the other studies done at home and abroad; the overall proportion of patients, in Ayder comprehensive specialized hospital, who responded for satisfaction with perioperative anesthesia service is low. Patient satisfaction level should be determined regularly and all bodies should work to decrease the factors which decrease the satisfaction level.

## Background

Patient satisfaction is a subjective and challenging perception, linking physical, expressive, psychological, societal, and cultural factors. Dissatisfaction happens if the patient feels an inconsistency between expected and delivered care. Because of the complexity and duration of the surgery, the pathophysiology of the disease is difficult to measure patient satisfaction with perioperative anesthesia care. Usually health care satisfactions is very high and according to many studies levels of satisfaction is above 85% and patient’s satisfaction in terms of anesthesia is not very different [1, 2].

Anesthetists now a days are considered to have greater participation in preoperative evaluation and postoperative care this should allow prior identification and treatment of postoperative adverse effects. They are expected to describe professional obligation of patient care which relate to direct anesthetist and patient interactions, he/she must earn respect and trust from the patients through detailed discussion on anesthetic plan and responding for any kind of concerns about the anesthesia [3–7]. Visiting the patient and discussing the perioperative issues of the anesthesia and patient concerns significantly enhance patient satisfaction, regardless of the type of anesthesia performed [7–12].

Of patients who have got general anesthesia and local anesthesia for different kinds of surgery, 87% of patient was satisfied, 0.5% dissatisfied and 12.5% had no opinion [13]. Whereas another study discovered that 21.54% of patients succeeded an overall satisfaction less than 85%, and female, and educated patients were less satisfied [14].

In Ethiopia, health coverage is inadequate and of poor quality, and the country has exceptionally poor health status compare to other low-income countries [15]. And dissatisfaction with anesthesia has been reported to be associated with a 12-fold risk of global dissatisfaction with day case surgery and, to the investigator’s knowledge, researches on elective inpatient satisfaction has rarely been reported in the literature [16–20]. Therefore we conducted this research to identify the satisfaction level of patients who have got surgery in Ayder Comprehensive Specialized Hospital and the result of this study will also use as baseline assessment for any further researchers and policy makers since there is not adequate studies on the subject area.

## Methods

### Study area, period and study design

Institution-based cross sectional-study design was used among patients who were scheduled for different kinds of surgery from surgical, orthopedics, medical, and gynecologic ward. The study was conducted from December 1/2017 to January 1/2018 at Mekelle University Ayder Comprehensive Specialized Hospital. Mekelle University Ayder Comprehensive Specialized Hospital is found in the Tigray regional state which is 780km far from the capital city of Ethiopia.

### Sample size and sampling procedure

Using consecutive sampling procedure all elective patients (120) from surgical, orthopedics, medical, and gynecologic ward that were operated upon under general or regional anesthesia during study were included.

### Data collection

Pre-tested structured questionnaire was prepared by reviewing previously done studies on the topic of a prospective study on elective surgical inpatient satisfaction with perioperative anaesthesia service [13, 14, 20]**.** The questionnaire was first prepared in English language by English language expert and then translated into Amharic by Amharic language expert, then to Tigrigna which is the local language of the patient in the study area by Tigrigna language expert. After the data has been collected the information acquired from the study participants changed to Amharic then to English by respective language experts.

Two data collectors who had experience on clinical data collection used after debriefing on the data collection methods by the principal investigators. Pretest on 10 patients was done before the actual data collection date and some of the terms were rephrased which were not clear for the participants. Data comprehensiveness and uniformity was checked by the investigators.

Data collection was done on socio-demographic characteristics and factors associated with perioperative anesthesia management of patients, satisfaction level and general satisfaction on perioperative anesthesia management after 24 h of operation using face to face interview. Before getting to the operation theater participants were educated about the standard care should provide for any patients on the context of anesthesia like detail information about the type of anesthesia the expected postoperative complications, having parts on the selection of anesthesia and others. After 24 h of their operation participants were asked to respond for whether they were involved on the decision of the type of anesthesia they could have, adequate information about the type of anesthesia they will have, had they any unnecessary disturbing events, whether they got relief for their any kind of discomfort on the postoperative time and level of their satisfaction and overall satisfaction.

### Data analysis

Epi Info version 6 was used to record the data and SPSS version 20 was used for the analysis. Descriptive statistics were used to explore the socio-demographic characteristics of patients; factors possibly related to satisfaction level and overall satisfaction were summarized as frequencies and percentages. Chi-square tests were employed. Variables associated with satisfaction of participants on perioperative anesthesia management in chi-square tests analysis were included and *P*-values less than 0.05 were considered to be statistically significant in all cases.

### Exclusion criteria

Patients were excluded if they have current history of mental illness, if he/she was operated previously in our hospital, critically ill (ASA 3–6) patient and patients who unable to communicate, patients who admitted to intensive care unit for further management, and pregnant patients were also excluded.

### Definition of terms

- **ASA 1**: A normal healthy patient.
- **ASA 2**: A patient with a mild systemic disease
- **ASA 3**: A patient with a severe systemic disease that is not life-threatening.
- **ASA 4**: A patient with a severe systemic disease that is a constant threat to life**.**
- **ASA 5**: A moribund patient who is not expected to survive without the operation. The patient is not expected to survive beyond the next 24 h without surgery.
- **ASA 6**: A brain-dead patient whose organs are being removed with the intention of transplanting them into another patient [21]

## Result

### Socio demographic characteristics of the participants

One hundred twenty consecutive patients were originally enrolled in the study that took over 1 month. None of them refused to fill out the questionnaire. As shown in Table 1 the baseline characteristics of the final 120 participants were active and 49.1% of them were male (Table 1).

**Table 1:** Socio demographic characteristics of surgical inpatients who has gone through surgery in Ayder comprehensive specialized Hospital, Mekelle, Ethiopia 2017/18

Characteristics	Category	Number	Percentage
Sex	Male	59	49.1%
	Female	61	50.9%
Payment status	Paying	107	89.16%
	Free	13	10.84%
Marital status	Single	32	26.7%
	Married	72	58.3%
	Divorced	8	7.00%
	Widowed	8	7.00%
Educational level	Primary school	89	74.17%
	Secondary school	25	20.83
	Higher education	6	5.00%

As described in Table 1, from these 120 patients, 100 of them received general anesthesia and 20 of them received regional anesthesia. Of the respondents 89 (94.17%) completed primary school and 6 (5%) had certificate for diploma or above. The Paying status of participants was asked in the preoperative time and 89.16% of the participants responded that they are categorized under paying patients for every service they got (Table 1).

### Association between satisfaction level and determinant factors

Chi-square test analysis was done to test the satisfaction level of participants with different parameters. General question that indicates the satisfaction rate of the patient on the anesthesia service was provided for the participants. From these participants106 (88.33%) of theme responded that they are satisfied on the anesthesia service. Furthermore, from the total patients who responded their satisfaction on perioperative anesthesia service only 56 (91.80%) of them were female. Then again, the male participants were 2 times more dissatisfy than the female participants though it is not statically significant (chi-square = 1.45, *P* = 0.2285). Participants were categorized in four group of age to test the association between the satisfaction level and age. As a result, the younger and the oldest participants were less satisfied on the perioperative anesthesia service than the middle age group which is statically significant (chi-square = 12.689, *P* = 0.0054).

Some Complication and specific tasks which are directly related to the type of anesthesia might decrease the satisfaction rate of the patient. The association indicates the type of the anesthesia and the satisfaction level. It was cheeked that patients who were operated under general anesthesia were 2 times more dissatisfied than who underwent their operation under regional anesthesia. However, the difference on the satisfaction level is not statically significant (chi-square = 2.733, *P* = 0.0983). Moreover, chi-square test analysis was also done to test the satisfaction level between participants who were visited and not visited by the anesthesia provider a day before the day of the operation. As a result, participants who were visited by the anesthesia provider were 4 times more satisfied than those who were visited by the anesthesia provider (chi-square = 27.102, *P* < 0.0001) which is statically significant. Education level is also one important factor for patient satisfaction and participants were classified in three groups to cheek the satisfaction level. Accordingly, the analysis shows that participants who has higher education were more dissatisfied than the other group which is statically significant (chi-square = 40.151, *P* < 0.0001) (Table 2).

**Table 2:** Association between different parameters versus satisfaction rate of surgical inpatients that had undergone surgery at Ayder comprehensive Specialized Hospital, Mekelle, Ethiopia 2017/18

Variable		Satisfied	Not satisfied	Chi square	*p*-value
Sex	Male	50	9	1.45	0.2285
	Female	56	5		
Age (Year)	15–24	11	7	12.689^a^	0.0054
	25-44	44	4		
	45–64	46	2		
	65+	5	1		
Education status	Primary	89	0	40.151 ^a^	< 0.0001
	Secondary	15	10		
	Higher	2	4		
Types of anaesthesia	General	91	9	2.733 ^a^	0.0983
	Regional	15	5		
Preoperative evaluation	Visited	104	8	27.102 ^a^	< 0.0001
	Not-visited	2	6		

^a^Yates corrected chi-square

Different perioperative complications, which might affect the satisfaction level, were put in to the chi-square analysis to test the value of clinical factors of patient satisfaction. The incidence rate of patients who had pain in perioperative time in general anesthesia were 2 times more than the participants who were operated in regional anesthesia group. Besides, the relationship between pain and satisfaction among the group in the chi-square test analysis is not strong (chi-square = 0.6047, *P* = 0.4374).

Dyspnea was also one of the concerns that was raised by the patient for the decrement of the satisfaction level. Subsequently, 18.75% of the patients who went through their operation under general anesthesia are dissatisfied; whereas all patients who were operated under regional anesthesia and who has dyspnea were satisfied. Nevertheless, chi-square test analysis shows that the difference between the group is not statically significant (chi-square = 0.0112, *P* = 0.7379). Nausea and vomiting was also another concern raised by the patients and these affect their satisfaction level. Thus, 50% of the patients who went through their operation under regional anesthesia were dissatisfied and 2.26% of the patients were from general anesthesia group. Then, chi-square test analysis shows strong relationship between nausea and vomiting and dissatisfaction among the group (chi-square = 6.331, *P* = 0.0119) (Table 3).

**Table 3:** Association between different perioperative complications versus satisfaction of surgical inpatients that had undergone surgery at Ayder comprehensive Specialized Hospital, Mekelle, Ethiopia 2017/18

Variable		Satisfied	Not satisfied	Chi-square	*P*- Value
Pain	Regional anesthesia	1	0	0.6047 ^a^	0.4374
	General anesthesia	11	3		
Dyspnoea	Regional anesthesia	2	0	0.112 ^a^	0.7379
	General anesthesia	13	3		
Shivering	Regional anesthesia	2	1	0.375 ^a^	0.5403
	General anesthesia	0	0		
Cold	Regional anesthesia	3	2	0.16 ^a^	0.6892
	General anesthesia	4	2		
Nausea and vomiting	Regional anesthesia	2	2	6.331^a^	0.0119
	General anesthesia	38	1		

^a^Yates corrected chi-square

**Fig. 1:**
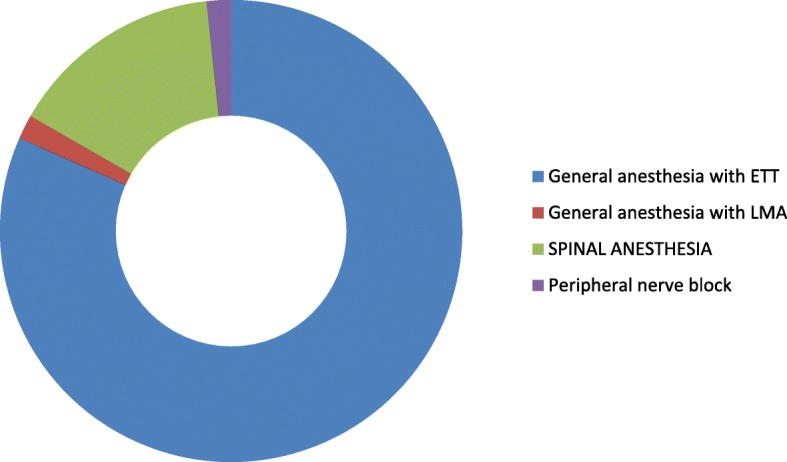
Types of anesthesia used for different kinds of surgery at Ayder comprehensive Specialized Hospital, Mekelle, Ethiopia 2017/18

**Fig. 2:**
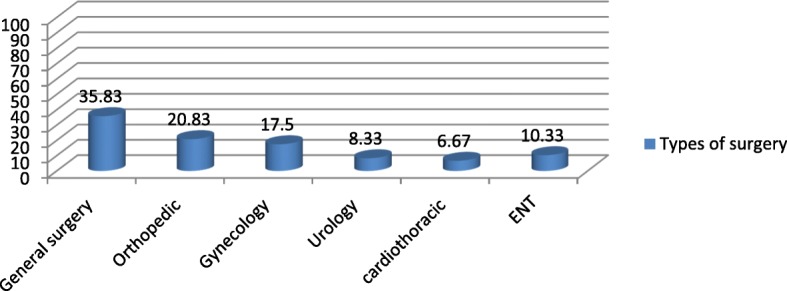
Shows the Types of procedure done at Ayder comprehensive specialized Hospital, Mekelle, Ethiopia 2017/18

**Fig. 3:**
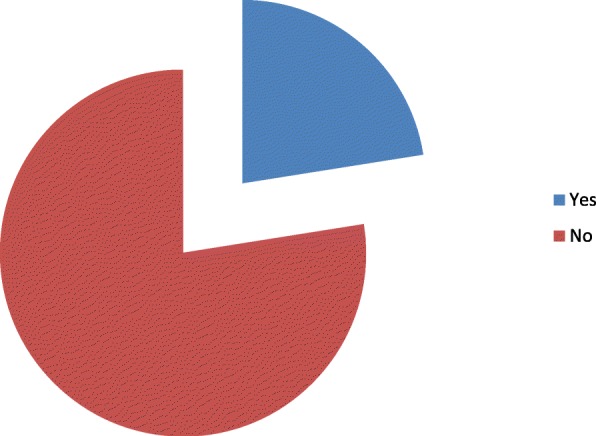
Presence of associated morbidity on patients those who got surgery at Ayder comprehensive specialized Hospital, Mekelle, Ethiopia 2017/18

**Fig. 4:**
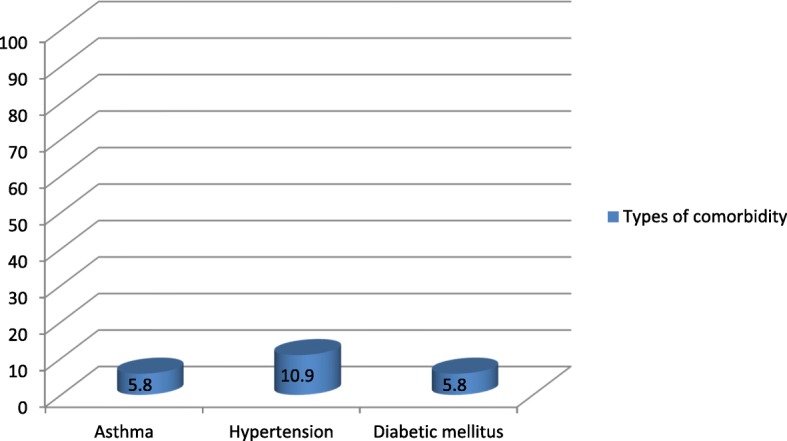
Types of associated morbidity on patients those who got surgery at Ayder comprehensive specialized hospital, Mekelle, Ethiopia 2017/18

**Fig. 5:**
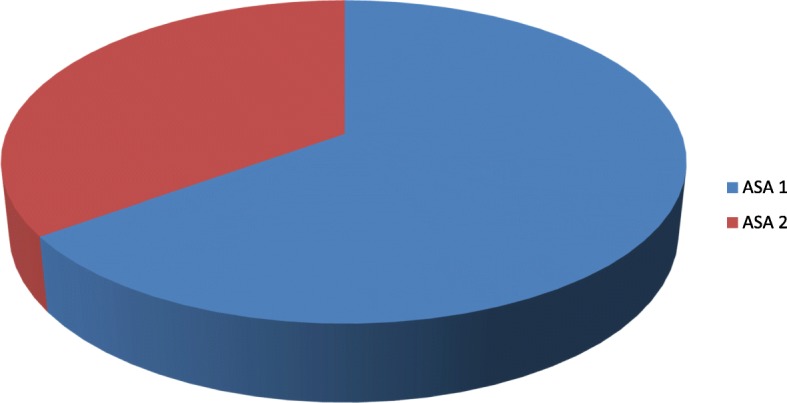
Shows physical status of patients who were operated at Ayder comprehensive Specialized Hospital Mekelle, Ethiopia 2017/18

**Fig. 6:**
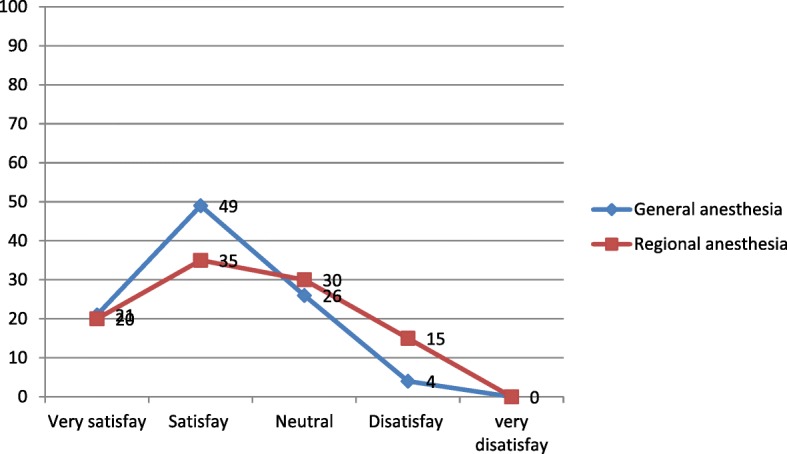
Shows level of satisfaction of patients those who undergo their operation by regional and general anesthesia at Ayder comprehensive specialized Hospital mekelle Ethiopia 2017/18

**Fig. 7:**
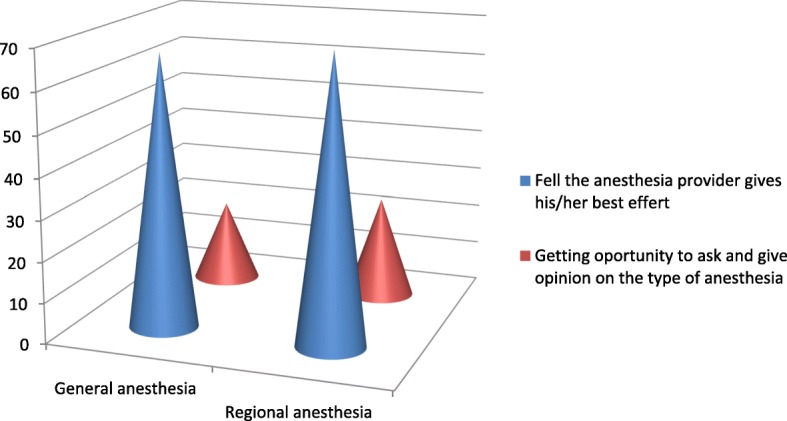
Shows the attitude of the patients towards the anesthesia provider and the whole stay when they undergo their operation by general and spinal anesthesia at Ayder comprehensive specialized Hospital Mekelle Ethiopia 2017/18

**Fig. 8:**
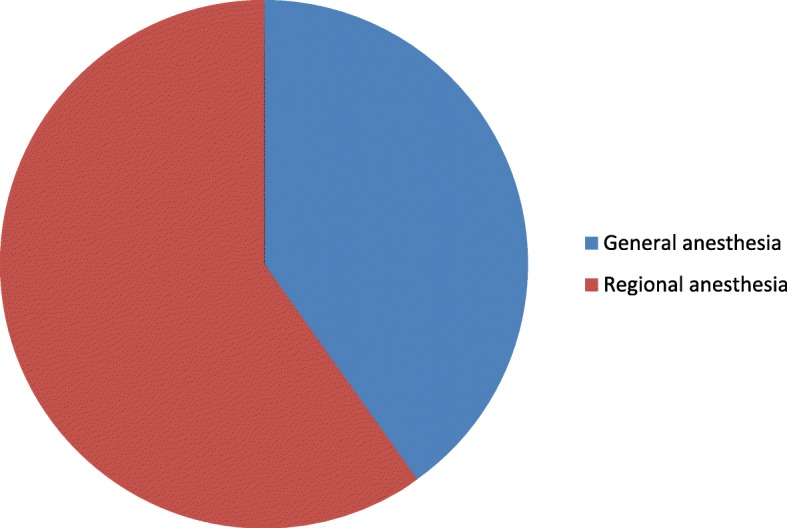
Shows desire of the participants on having the same kinds of anesthesia if they have the same kinds of surgery at Ayder comprehensive specialized hospital Mekelle Ethiopia 2017/18

 The types of anesthesia was also analyzed where the majority which is 100 (83.33%) of the patients had got general anesthesia. Moreover, 98 (98%) was operated under general anesthesia with endotracheal tube (ETT). Conversely, the remaining 2 (2%) was operated under general anesthesia with laryngeal mask airway (LMA) (Fig. 1). Types of surgery was also recorded and from 120 patients 43 (35.83%) were general surgery cases, 25 (20.83%) orthopedic cases, 8 (6.67%) cardiothoracic cases, 13 (10.83%) ENT cases, 21 (17.50%) gynecology cases and 10 (8.33%) urology cases (Fig. 2). Among the 120 patients who undergone surgery, 27 (22.5%) were associated to co-morbidities and of which 7 (5.83%) were asthmatic, 13 (10.83%) were hypertensive, and 7(5.83%) were diabetic patients shown in figure 3 and figure 4 respectively) (Figs. 3 and 4). Using the America Society of Anesthesiologist (ASA) standard, the overall health status of the patients was determined. Thus, of the total 78 (65%) were ASA 1 patients and 42 (35%) were ASA 2 patients (Fig. 5). Using Likert scale all patients were asked for the degree of satisfaction they had at recovery room when they are ready for transfer to their ward; 25 (21%) and 4 (20%) of the participants responded they are very satisfied, 56 (49%) and 7 (35%) responded satisfied, 32 (26%) and 6 (30%) responded neutral, 7 (4%) and 3 (15%) participants responded that they are dissatisfied on anesthesia service they have got from general and spinal anesthesia respectively. From both groups no participant response for very dissatisfied (Fig. 6). The professional approach to the patient which is getting consent of the patient for any kind of procedure is done. And this includes the decision of the anesthesia management in which it significantly affects the anesthetic satisfaction rate; participants were asked whether they think that they get best help from their anesthesia provider. Hence, 67 (67%) of them are from the general anesthesia group and 14 (70%) of them are from the regional anesthesia group who felt that the anesthesia provider did best for them. And similarly, they were asked whether the anesthesia provider got consent for any procedure done to the participant and did they get the opportunity to ask questions and give opinion on the anesthetic management. Accordingly, 20 (20%) from general anesthesia group and 5 (25%) from regional anesthesia group responded that they had discussion and gave their opinion on the type of anesthesia they have got (Fig. 7). Specific question was provided which address the desire of the participant on having the same kind of anesthesia management by the same anesthetist if they have the same kind of operation. Thus 55 (47%) participants from the general anesthesia group and 14 (70%) participants from the spinal anesthesia group want to have the same kind of anesthesia intervention and management (Fig. 8).

## Discussion

Our study showed that the overall proportion of participants who said we were satisfied with perioperative anesthesia services in Mekelle University Ayder Comprehensive Specialized Hospital was 88.33%. This finding was low compared with other studies done abroad and home [4, 18, 19, 22]. This can be secondary to poor preoperative assessment because the patient was not included on the selection of anesthesia type, and there was poor perioperative patient handling. On the other hand, some reference studies show that the technique of anesthesia was general anesthesia with patient controlled analgesia. And that minimizes the postoperative pain incidence and maximizes the satisfaction rate. And another reason for the low satisfaction level in our study is nausea and vomiting (39 and 20%), difficulty to breath in the immediate postoperative time (16 and 10%) and pain (14 and 5%) were the factors which had high percentage form factors which can decrease the satisfaction level in general and regional anesthesia respectively.

In this study the extreme age group (15–24 yrs. and 65+ yrs.) were less satisfied than the middle age groups (*P* = 0.0054), the result of this study matches with study done in Gonder University Hospital [20]. Patient dissatisfaction rate was high at the age of 20–39 years old. But the result of our study did not match with study done in Japan. This difference might be due to small number of patients were included in mentioned age group in Japan’s study [23].

According to some studies, satisfaction rate is affected by the sex of the participants where female participants are less satisfied than male [1, 20]. But in this study female participant are more satisfied than male which was 91.80 and 84.75% respectively (*P* = 0.2285). This difference could be explained by the duration of stay in the reference study [1, 20] which was very short. Moreover, the type of surgery in our study was not life threating and preoperative hospital stay was not long for female patients. And this is because the waiting list for surgery from female side is not that much.

In our study, those who were more educated had low level of satisfaction. 100 and 33.33% of participants had a maximum of primary school and went for higher education respectively (*P* < 0.0001). The result of our study matches with study done in Saudi Arabia which showed that the educated participants had low satisfaction level [14].

In this study, less participants who were operated under regional anesthesia (75%) were satisfied than participants operated upon general anesthesia (general vs regional, *P* = 0.0983). But the study conducted at University of Gonder Hospital [20] indicate that participants who were operated under regional anesthesia was satisfied than those who were operated under general anesthesia. This discrepancy might be explained as some of patients in our study had peripheral block which usually cause pain on multiple attempts.

In our study participants who were evaluated by the anesthesia provider in the preoperative time had high satisfaction level (92.86%) than those who didn’t get the anesthetist visit a day before the surgery (*P* < 0.0001). This result is in line with a study done in Australia [24]; this could be because of reassurance of patients and knows more about types of anesthesia that they will have.

### Limitation of the study

Due to time constraint, small sample size was the limitation of the study. And this is because of the short time devoted between anesthesia and assessment of satisfaction.

## Conclusion

Compared with the other studies done at home and abroad; the overall proportion of patients, in Ayder comprehensive specialized hospital, who responded for satisfaction with perioperative anesthesia service is low.

## Recommendations

Patient satisfaction level should be done regularly at least once in a year. The official bodies of the hospitals, the anesthesia providers, nurses who work at ward and post anesthesia care unit have great responsibility to solve the precipitating factors for the low satisfaction level. In addition, the anesthesia provider should minimize the dissatisfying factors and increase factors exploiting satisfaction to reach at the final endpoint of patient satisfaction and quality of health service delivery. Training should also be given to anesthetists in the areas of the communication skills.

## Abbreviations

ASA: America society of anesthesiology
BMI: Body mass index
ENT: Ear, nose and throat
ETT: Endotracheal tube
LMA: Laryngeal mask airway
LMP: Last menstrual period
MDGs: Millennium Development Goals
PONV: Post operative nausea and vomiting
SPSS: Statistical package for social sciences

## References

[1] Auquier P, Pernoud N, Bruder N (2005). Development and validation of a perioperative satisfaction questionnaire. Anesthesioloy.

[2] Fung D, Cohen M (1998). Measuring patient satisfaction with anaesthesia care: a review of current methodology. Anesth Analg.

[3] Heppner DL, Badner AM, Hurwitz S, Gustatson M, Tsen LC (2004). Patient satisfaction with preoperative assessment in a preoperative assessment testing clinic. Anesthesia Analg.

[4] Evaluation of Patient Satisfaction and Recovery Time Following Different Anesthetic techniques at the San Ignacio University Hospital. Rev Col Anesthesia. 2010;38(2):178–202.

[5] Gill TM, Feinstein AR (1994). A critical appraisal of the quality-of-life measurements. JAMA.

[6] Abbey A, Andrews FM (1985). Modelling the psychosocial determinants of life quality. Soc Indic Res.

[7] Klock PA, Roizen MF (1996). More or better—educating the patient about the anesthesiologist’s role as perioperative physician. Anesth Analg.

[8] Lee A, Lum ME (1996). Measuring anaesthetic outcomes. Anaesth Intensive Care.

[9] Vyhunthan G, Aeshana de Silva NG. Audit to evaluate preoperative visit to patient by anaesthetist. Sri Lankan Journal of Anaesthesiology. 2012;20(2):88–91.

[10] Kouki et al, Greek surgical patients satisfactiondevopress. Patient Prefer Adherence. 2012:6:569–578. 10.2147/PPA.S34244 PMC3422116 22927750

[11] Yucelt U (1994). An investigation of causes of patient satisfaction/dissatisfaction with physician services. Health Mark Q.

[12] Tielsch JM, Steinberg EP, Cassard SD (1995). Preoperative functional expectations and postoperative outcomes among patients undergoing first eye cataract surgery. Arch Ophthalmol.

[13] Wołowicka L (2001). Patient satisfaction with anesthesia as a measure of quality of anesthesia care. Folia Med Cracov.

[14] Baroud ND, Nofal HW, Ahmad AN (2010). Patient satisfaction in anesthesia. A modified Iowa satisfaction in anesthesia scale. Anesth Essays Res.

[15] Girma S (2007). Human resource development for health in Ethiopia: challenges of achieving the millennium development goals. Ethiop J Health Dev.

[16] Myles PS, William DL, Handratam M, Anderson H, Weeks AM (2000). Patient satisfaction after anesthesia and surgery; result of a prospective surgery of 10811 patients. Br J Anesthesia.

[17] Kouki P, Matsota P, Christodoulaki K (2012). Greek surgical patients’ satisfaction related to preoperative anesthetic services in an academic institute. Patient Prefer Adherence.

[18] Lehman M (2010). Postoperative patient compliant prospective interviews study of 12276 patients. J Ansth Clin.

[19] Belihun A, Alemu M, Mengistu B. A Prospective Study on Surgical Inpatient Satisfaction with Perioperative Anesthetic Service 2015 6; 3 8.

[20] Gebremedhn E, et al. Patient satisfaction with anesthesia with anaesthesia services and associated factors at the University of Gondar Hospital. 2013;8:377. https://www.ncbi.nlm.nih.gov/pmc/articles/PMC4549915/10.1186/s13104-015-1332-4 PMC4549915 26306394

[21] DJ Doyle; Emily American Society of Anesthesiologists Classification (ASA Class) October 6, 2017. https://www.ncbi.nlm.nih.gov/books/NBK441940.

[22] Fernandez MB (2013). Assessing patient satisfaction with cataract surgery under topical anesthesia supplemented by intracameral lidocain combined with sedation. Arq Bras Oftalmol.

[23] Nakahashi K, Motozu Y, Sasaoka N, Hirai K, Kitaguchi K, Furuya H (2004). Patient satisfaction with anesthesia care. Masui..

[24] Pollard JR, Coyle PJ, Gilbert LR, Beck EJ (2007). Intraoperative awareness in a regional medical system. A review of 3 years’ data. Anesthesiology.

